# A combination of plasma DAO and citrulline levels as a potential marker for acute mesenteric ischemia

**DOI:** 10.3402/ljm.v8i0.20596

**Published:** 2013-03-26

**Authors:** Rıdvan Çakmaz, Oktay Büyükaşık, Nurettin Kahramansoy, Hayri Erkol, Cavit Çöl, Çetin Boran, Güler Buğdaycı

**Affiliations:** 1 Department of General Surgery, State Hospital, Adıyaman, Turkey; 2 Department of General Surgery, Lokman Hekim Hospital, Sincan, Turkey; 3 Department of General Surgery, Faculty of Medicine, Abant Izzet Baysal University, Bolu, Turkey; 4 Department of Pathology, Faculty of Medicine, Abant Izzet Baysal University, Bolu, Turkey; 5 Department of Biochemistry, Faculty of Medicine, Abant Izzet Baysal University, Bolu, Turkey

**Keywords:** animal model, experimental, intestines, diamine oxidase, citrulline

## Abstract

**Introduction:**

There is no valid and reliable diagnostic test for early diagnosis of acute mesenteric ischemia (AMI). The aim of this study was to measure the plasma levels of diamine oxidase (DAO) and citrulline in AMI to gain insight into its early diagnosis.

**Material and methods:**

A total of 21 Wistar albino rats were divided into three groups, that is, control group, short-term ischemia group, and prolonged ischemia group. The superior mesenteric artery was occluded for 15 min in the short-term ischemia group and for 12 h in the prolonged ischemia group. Twelve hours later, the experiment was terminated and plasma DAO and citrulline levels were measured. Intestinal tissue was evaluated for the histopathological changes.

**Results:**

Compared to the control group, the short-term and prolonged ischemia groups showed significant increases in the plasma levels of DAO, whereas the plasma citrulline levels decreased significantly. Prolonged ischemia caused a larger increase in the plasma DAO levels and a larger decrease in the plasma citrulline levels compared to the short-term ischemia (*p=*0.011 and *p*=0.021, respectively). Intestinal damage was shown to develop more in the prolonged ischemia group (*p=*0.001).

**Conclusion:**

In the early period of AMI, the plasma DAO levels increase while citrulline levels decrease, and the extent of these changes depends on the duration of ischemia.

Acute mesenteric ischemia (AMI) is a serious pathology that causes acute abdomen and is life-threatening. AMI mostly occurs in the elderly or in patients with cardiac problems (1–3). Its incidence is higher than 10 in 100,000 (4). Despite the recent improvements in diagnostic and therapeutic methods and postoperative intensive care practices, the morbidity and mortality associated with AMI remain quite high (2, 3). Among many factors that affect AMI management, the most important is early diagnosis and early revascularization (5). For this reason, early diagnosis of AMI is of great importance. Various methods have been studied for the early diagnosis of AMI, but no diagnostic test has been proven to be reliable with high sensitivity and specificity (6–10).

Diamine oxidase (DAO) is an enzyme that catalyzes oxidative deamination of histamine-like diamines. It is mainly produced in the small intestine, where it is found in high amounts (11–13). It is reported that DAO levels in plasma increase in cases of intestinal ischemia, inflammation, and similar stresses (14–16). Citrulline is an amino acid that is produced mainly in enterocytes from glutamate (17). The plasma citrulline level decreases in various cases when intestinal capacity and mucosal barriers are damaged (18–20). The aim of this study was to measure the plasma DAO and citrulline levels in the early period of AMI and to evaluate the utility of this information as a potential marker for the early diagnosis of AMI.

## Material and methods

The present study was conducted in an Experimental Research Center with the permission of University Local Ethics Committee of Research on Experimental Animals (2010/25). A total of 21 female Wistar albino rats weighing 250–300 g were used in this experiment. All of the animals were housed and fed under physiological conditions in accordance with the international regulations and guidelines. Rats were randomly assigned to three groups with seven rats in each. The control group, the short-term ischemia group, and the prolonged ischemia group were formed randomly, assigning seven rats to each group. For general anesthesia, 50 mg/kg ketamine and 10 mg/kg xylazine were injected intramuscularly. Surgical interventions were performed under standard sterile conditions through a midline incision.

### Control group

The surgical procedure was terminated after exploration of the superior mesenteric artery.

### Short-term ischemia group

The superior mesenteric artery was explored and occluded by a Bulldog Clamp for 15 min. At the end of the occlusion period, the clamp was released, and reperfusion was enabled. The abdominal wall was closed primarily with 3-0 silk suture.

### Prolonged ischemia group

The superior mesenteric artery was explored and ligated (occluded) using a 3-0 silk suture. The abdominal wall was then closed primarily with 3-0 silk suture.

Relaparotomy was performed in all groups after 12 h following the surgical intervention. The animals were sacrificed by cardiac puncture. Blood samples were used for biochemical analysis. A 10 cm ileum segment was resected for histopathological investigation. The blood samples were put in citrated tubes, and the intestinal tissues were fixed in a solution of 10% formaldehyde. Plasma urea and creatinine levels were measured in order to assess renal function, because the plasma citrulline level is reported to increase as a result of its lack of clearance in renal dysfunction (17, 21).

### Biochemical evaluation

For the measurement of the plasma DAO level, Rat Diamine Oxidase ELISA kit was used (Catalog no: CSB-E12634r, Cusabio Biotech. Wuhan). For the measurement of plasma citrulline level, Rat Citrulline ELISA kit was used (Catalog no: CSB-E13414r, Cusabio Biotech. Wuhan). Plasma DAO and citrulline levels were measured with a spectrophotometric method.

### Histopathological investigation

The ileum was embedded in paraffin. Tissue sections of 5 µm thickness were stained with hematoxylin-eosin (H-E). Morphological changes were evaluated by using a scoring system as *Grade 1:* normal appearance of intestinal tissue, *Grade 2:* minimal hydropic degeneration in surface epithelium, *Grade 3:* minimal necrosis of the surface epithelium at the tip of the villus, *Grade 4:* necrosis at the tip of the villi, *Grade 5:* full thickness mucosal necrosis, and *Grade 6:* transmural necrosis (22).

### Statistical analysis

Statistical analysis was done using SPSS 17.0 statistical package. Results are reported as mean±standard deviation (SD). Kruskal Wallis test was used for comparison of all three groups. The binary comparison of the groups was performed using Mann Whitney U test. Statistically significance was considered as *p*<0.05.

## Results

During the experiment no mortality was observed in any of the groups. Blood urea and creatinine levels were normal in all groups. Changes in the plasma citrulline level were evaluated making sure that there is no change resulting from renal impairment (Table 1).

**Table 1:** Plasma urea, creatinine, DAO (diamine oxidase), and citrulline levels in groups and the comparisons among the groups

	Control	Short-term ischemia	Prolonged ischemia	*p*
Urea (mmol/L)	13.89±2.20	15.88±1.73	15.22±2.32	0.22
Creatinine (µmol/L)	47.28±2.28	46.51±6.10	43.46±7.62	0.47
DAO (IU/L)	0.77±0.18	3.15±0.42	3.92±0.55	0.0028
Citrulline (µmol/L)	53.00±4.20	23.85±4.52	18.42±2.69	0.0037
Histopathologic score	1.71±0.75	3.57±0.97	5.00±0.81	0.001

Mean±standard deviation.

Mean plasma DAO level was 0.77±0.18 mIU/mL in the control group, 3.15±0.42 mIU/mL in the short-term ischemia group, and 3.92±0.55 mIU/mL in the prolonged ischemia group. The difference of the mean plasma DAO levels among the groups was statistically significant (*p*=0.0028). Binary comparisons also revealed the significance of the differences between the control group and each of the ischemia groups (*p*=0.002) as well as between the two ischemia groups (*p*=0.011). Plasma DAO increased to a higher level in the rats subjected to the longer duration of AMI.

Mean plasma citrulline values were 53.00±4.20 µmol/L in the control group, 23.85±4.52 µmol/L in the short-term group, and 18.42±2.69 µmol/L in the prolonged ischemia group. The differences among the groups were statistically significant (*p*=0.0037). The mean plasma citrulline values were also significantly different between pairs of groups: control–short-term groups (*p*=0.002), control–prolonged ischemia groups (*p*=0.002), and short-term–prolonged ischemia groups (*p*=0.021). The longer the duration of AMI, the greater the decrease in plasma citrulline level.

### Histopathological evaluation

**Fig. 1:**
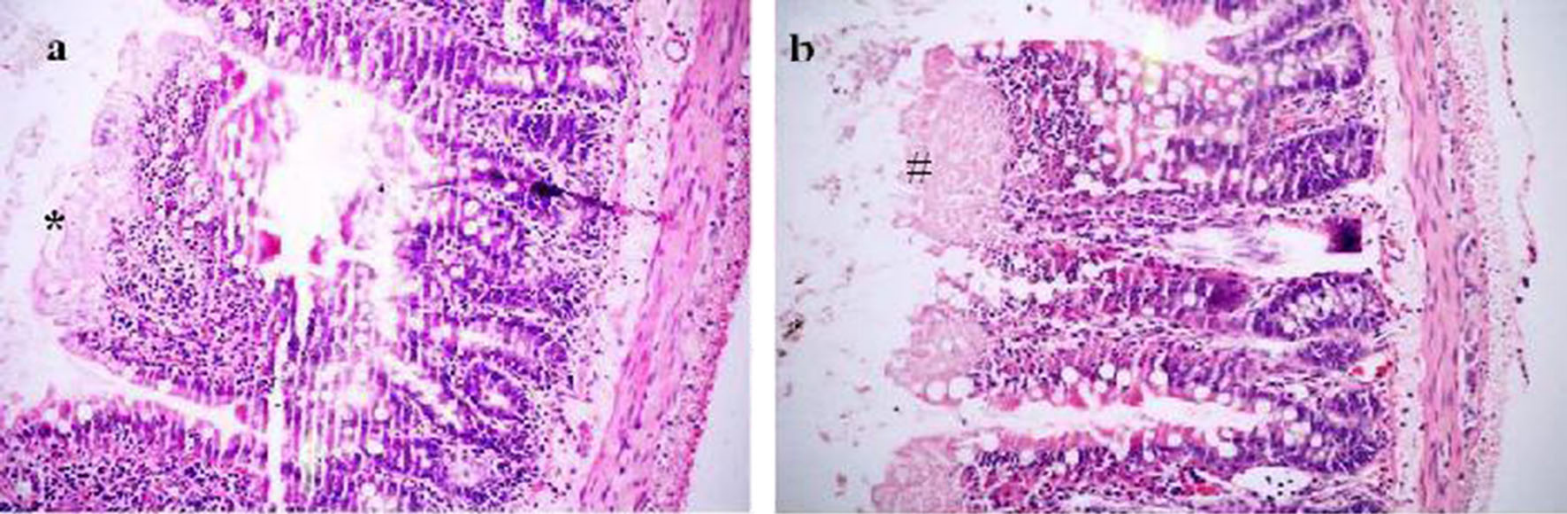
Morphological changes of the intestine in the short-term ischemia group. Panel a demonstrates the score Grade 3 (*: minimal necrosis of the surface epithelium at the tip of the villus). Panel b shows the score Grade 4 with (#) necrosis at the tip of the villi (H-E, ×200).

**Fig. 2:**
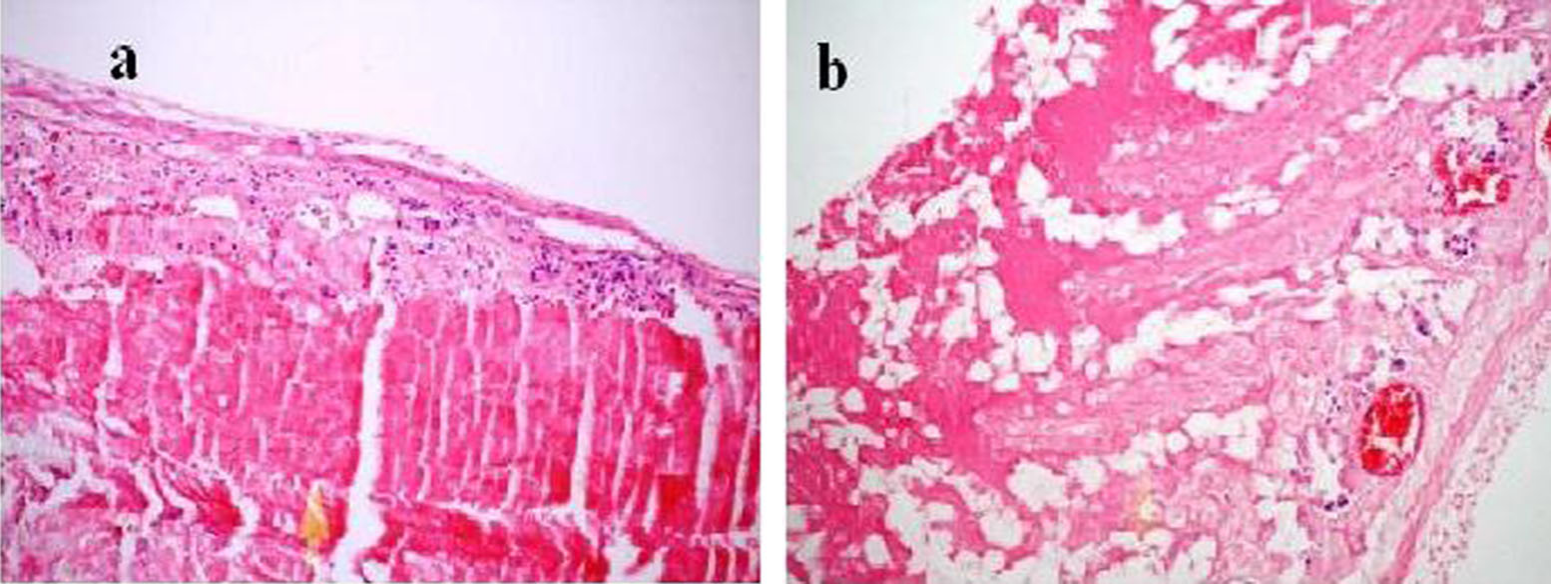
Morphological changes of the intestine in the prolonged ischemia group. Panel a demonstrates the score Grade 5 with full thickness mucosal necrosis. Panel b shows the score Grade 6 with transmural necrosis (H-E, ×200).

Intestinal ischemia was observed macroscopically only in the prolonged ischemia group. In the short-term ischemia group, the microscopic evaluation of the ischemia revealed that Grade 2, 3, 4, and 5 had progressed in 1, 2, 3, and 1 subjects, respectively (Fig. 1a, b). The prolonged ischemia group sustained more severe damage as Grade 4, 5, and 6 in 2, 3, and 2 subjects (Fig. 2a, b). There was a significant difference among the three groups (*p*=0.001) (Table 1). The binary comparison of the groups showed significant differences [control–short-term groups (*p*=0.006), control–prolonged ischemia groups (*p*=0.001), and short-term–prolonged ischemia groups (*p*=0.017)].

## Discussion

Mesenteric ischemia causes a hypoxic cell degeneration process in intestinal epithelial cells (23). This process disrupts the mucosal barrier and allows the entry of digestive enzymes into the intestinal wall, leading to autodigestion of the intestinal structure (24). In the case of reperfusion after mesenteric ischemia, intestinal damage is known to be caused by more than the ischemia alone (25). Various factors (essentially reactive oxygen species and activated neutrophils) during the reperfusion contribute to the intestinal injury (26). In our study, the experimental models of the short-term ischemia group and the prolonged ischemia group are different. However, the expected effect of the short ischemia is similar because the short time ischemia (15 min) causes slight intestinal injury that is not altered but is worsened insignificantly by reperfusion (23–25). This slight intestinal damage is sufficient for our comparison.

Many factors affect prognosis in AMI and increase morbidity and mortality, including the duration of ischemia, presence of shock or sepsis, fluid-electrolyte disorders, old age, cardiac, renal or respiratory diseases, and diabetes mellitus. Prognosis is also affected by generalized peritonitis, length of the remnant intestine, presence of colonic ischemia and resection, duration of operation and whether reoperation was conducted or not (1–3, 27). Due to all these factors, the mortality rate from AMI has been reported to be very high (3).

Early diagnosis and treatment of AMI are the most important factors in prognosis. For this reason, many tests were investigated for the diagnosis of the AMI. Some of the parameters are white blood cell count in blood, AST, ALT, LDH, ALP, CRP, CK-MB, D-dimer, troponin I, α-glutathione *S*-transferase, lactate, procalcitonin, and phosphorus levels in plasma (6–10, 28). Presently, angiography appears to have the highest sensitivity. However, it is not widely used because of its invasive nature and the difficulty of defining its indication early in the course of AMI. Therefore, many experimental and clinical studies are performed in search of a highly sensitive and non-invasive test for the early diagnosis of AMI. Plasma DAO level has been suggested to be one of the potential early markers which may be useful in the diagnosis of AMI (14, 15). Plasma DAO level is also reported to increase due to the intestinal inflammation, ischemia, or alteration of the intestinal mucosal integrity (11–15, 29). Wollin et al. reported that an experimental mesenteric ischemia for an hour caused the DAO level to decrease in the intestinal mucosa, and to increase in the intestinal lumen, mesenteric lymph nodes, and plasma (29). Bragg et al. reported that the increase of the DAO level in the intestinal lumen correlates with the duration of mesenteric ischemia (14). Currently, DAO measurement is frequently used in experimental studies as an extra supporting marker of intestinal damage with a specificity of 100%, accuracy of 95%, and sensitivity of 94% (16, 30–32). Tsunooka et al. analyzed the coronary bypass grafting of patients in whom cardiopulmonary bypass was either performed or not performed (33). They observed high plasma DAO levels in relation to the cardiopulmonary bypass, suggesting intestinal ischemia and damage progression. In our study, we found a 4–5 fold increase in the DAO level in the plasma of the rats with AMI compared to the control rats. Moreover, the plasma DAO level was significantly higher in the prolonged AMI group compared with the short-term AMI group. Our data demonstrate a strong association between the duration of the intestinal ischemia, the rise in plasma DAO level, and the severity of tissue damage.

In our study, we also addressed the relation between the plasma citrulline level and acute intestinal ischemia, about which we had found no published reports. Citrulline is an amino acid synthesized from glutamate in enterocytes and metabolized in the renal pathways. Therefore, the plasma citrulline level is expected to increase with renal dysfunction (17, 21). However, the plasma citrulline level has been shown to decrease secondary to reduction of intestinal functional capacity and impairment of the mucosal barrier (19, 20, 34, 35). The plasma citrulline level decreases significantly in patients with short bowel syndrome or due to massive resections of the intestine (18, 34, 35). Jiang et al. reported that efficiency of the rehabilitation treatment for short bowel syndrome could be evaluated by monitoring the change in plasma citrulline level (35). An increase in plasma citrulline level is suggested to be a marker of adequate intestinal length and absorption surface area (35). Also, in intestinal transplantation patients, the plasma citrulline level was found to decrease more during acute rejection compared to patients without rejection (36, 37). In another study, a high dose of chemotherapy was reported to decrease the plasma citrulline level as a consequence of the impairment of the intestinal mucosal barrier (20). In two other studies, intestinal mucosal damage was investigated by comparing plasma citrulline levels in patients with pelvic malignancy before and after radiotherapy. It was found that the plasma citrulline level decreased significantly following radiotherapy (38, 39). Moreover, plasma citrulline levels have been shown to decrease significantly, suggesting intestinal damage in patients secondary to pancreatitis or burns, and in patients in intensive care unit (ICU) (40, 41). Piton et al. detected low plasma citrulline levels in 44% of ICU patients and stated that the plasma citrulline measurement could be a sensitive marker for the functional enterocyte mass (42). Noordally et al. also studied intestinal dysfunction in ICU patients and found that among several parameters (SOFA-APACHE scores, CRP, pre-albumin, albumin, citrulline, inotrophic agents, and renal dysfunction) only low plasma citrulline level correlated well with intestinal dysfunction (43). As a different perspective, Yi et al. reported that exogenously administered L-citrulline exhibits gastric protection by the inhibition of neutrophil infiltration, which might be related in prevention of the increase in iNOS activity in an experimental gastric ischemia reperfusion study (44).

A plasma citrulline level below 27 µmol/L is thought to suggest intestinal pathology, and a level below 15 µmol/L is accepted as a marker of severe intestinal damage (18, 36, 39, 43). These reports suggest that the plasma citrulline level may be useful in the early detection of intestinal damage.

In our study, the plasma citrulline levels were significantly decreased in intestinal ischemic rats. Furthermore, the plasma citrulline levels decreased more with prolongation of ischemia and severity of the intestinal damage.

## Conclusion

The plasma DAO level increases and the plasma citrulline level decreases in AMI. Prolonged intestinal ischemia causes a greater increase in the plasma DAO level and a larger decrease in the plasma citrulline level. Therefore, a combination of the plasma DAO and citrulline levels seems to be a good marker for the early detection of AMI. However, extensive clinical studies are needed to corroborate these findings in humans.
